# Hydrogen Production and Enzyme Activities in the Hyperthermophile *Thermococcus paralvinellae* Grown on Maltose, Tryptone, and Agricultural Waste

**DOI:** 10.3389/fmicb.2016.00167

**Published:** 2016-02-23

**Authors:** Sarah A. Hensley, Emily Moreira, James F. Holden

**Affiliations:** Department of Microbiology, University of Massachusetts AmherstAmherst, MA, USA

**Keywords:** hyperthermophile, *Thermococcus*, hydrogenase, waste remediation, bioenergy

## Abstract

*Thermococcus* may be an important alternative source of H_2_ in the hot subseafloor in otherwise low H_2_ environments such as some hydrothermal vents and oil reservoirs. It may also be useful in industry for rapid agricultural waste treatment and concomitant H_2_ production. *Thermococcus paralvinellae* grown at 82°C without sulfur produced up to 5 mmol of H_2_ L^−1^ at rates of 5–36 fmol H_2_ cell^−1^ h^−1^ on 0.5% (wt vol^−1^) maltose, 0.5% (wt vol^−1^) tryptone, and 0.5% maltose + 0.05% tryptone media. Two potentially inhibiting conditions, the presence of 10 mM acetate and low pH (pH 5) in maltose-only medium, did not significantly affect growth or H_2_ production. Growth rates, H_2_ production rates, and cell yields based on H_2_ production were the same as those for *Pyrococcus furiosus* grown at 95°C on the same media for comparison. Acetate, butyrate, succinate, isovalerate, and formate were also detected as end products. After 100 h, *T. paralvinellae* produced up to 5 mmol of H_2_ L^−1^ of medium when grown on up to 70% (vol vol^−1^) waste milk from cows undergoing treatment for mastitis with the bacterial antibiotic Ceftiofur and from untreated cows. The amount of H_2_ produced by *T. paralvinellae* increased with increasing waste concentrations, but decreased in *P. furiosus* cultures supplemented with waste milk above 1% concentration. All mesophilic bacteria from the waste milk that grew on Luria Bertani, Sheep's Blood (selective for *Staphylococcus*, the typical cause of mastitis), and MacConkey (selective for Gram-negative enteric bacteria) agar plates were killed by heat during incubation at 82°C. Ceftiofur, which is heat labile, was below the detection limit following incubation at 82°C. *T. paralvinellae* also produced up to 6 mmol of H_2_ L^−1^ of medium when grown on 0.1–10% (wt vol^−1^) spent brewery grain while *P. furiosus* produced < 1 mmol of H_2_ L^−1^. Twelve of 13 enzyme activities in *T. paralvinellae* showed significant (*p* < 0.05) differences across six different growth conditions; however, methyl viologen-dependent membrane hydrogenase activity remained constant across all media types. The results demonstrate the potential of at least some *Thermococcus* species to produce H_2_ if protein and α-glucosides are present as substrates.

## Introduction

**Figure 1:**
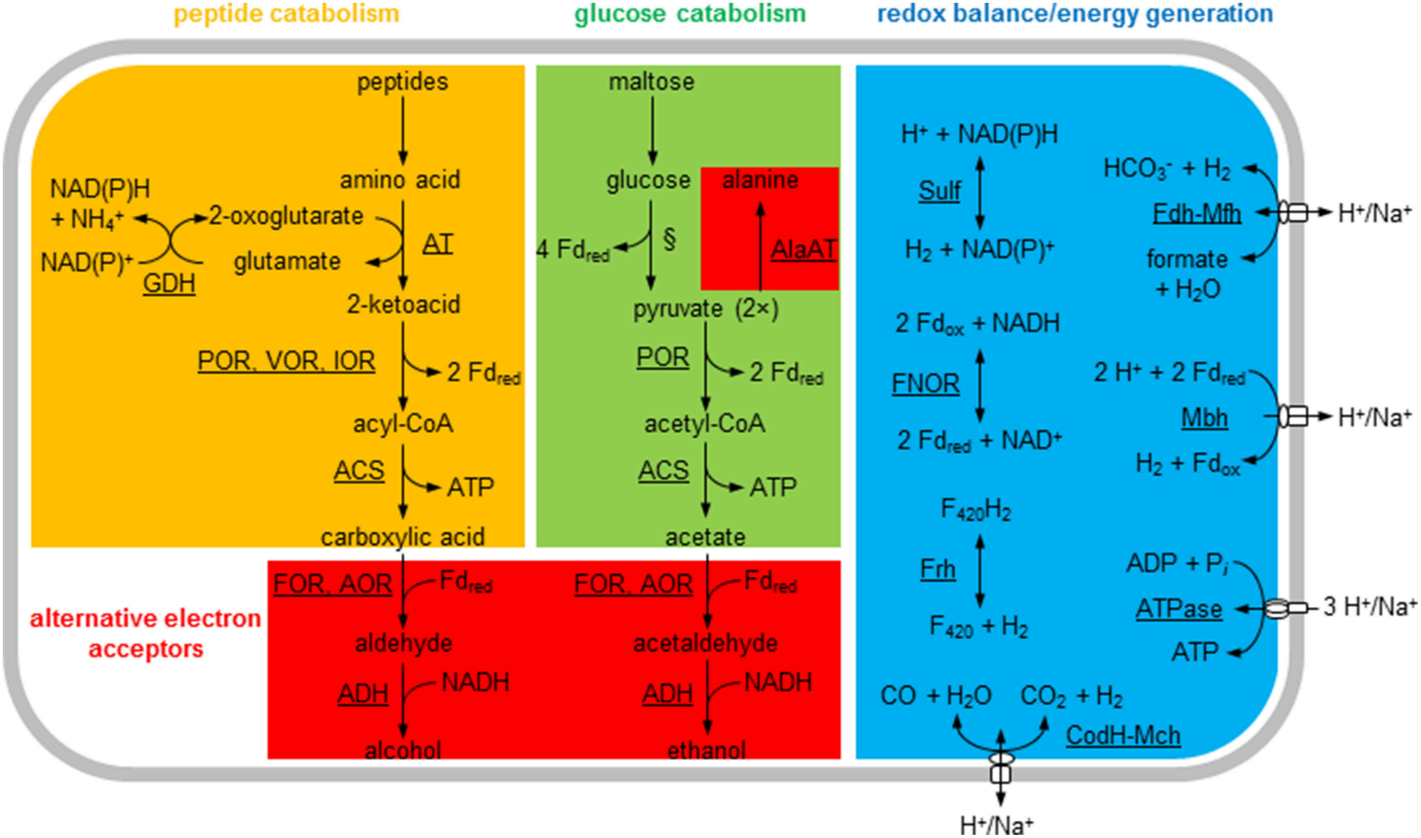
**General metabolic pathways for ***Thermococcus paralvinellae*****. The enzymes are membrane-bound hydrogenases: Fd-dependent (Mbh), formate-dependent (Mfh), and CO-dependent (Mch); cytoplasmic hydrogenases: NADH-dependent (Sulf) and F_420_-dependent (Frh); NADH:Fd oxidoreductase (FNOR); membrane-bound ATP synthase (ATPase); glutamate dehydrogenase (GDH); alanine aminotransferase (AlaAT); general aminotransferases (AT); pyruvate:Fd oxidoreductase (POR); isovalerate:Fd oxidoreductase (VOR); indolepyruvate:Fd oxidoreductase (IOR); ADP-forming acetyl-CoA/acyl-CoA synthetase (ACS); aldehyde:Fd oxidoreductase (AOR); formaldehyde:Fd oxidoreductase (FOR); and alcohol dehydrogenase (ADH). Fd, electron carrier ferredoxin; F_420_, electron carrier coenzyme F_420_; §, modified Embden-Meyerhof pathway. The activities for most of the enzymes listed are shown in **Table 4**.

*Thermococcus* species are hyperthermophilic heterotrophic archaea that catabolize carbohydrates and peptides and produce organic acids and CO_2_ as metabolites (Figure 1; Adams, 1999). Their growth is often associated with sulfur reduction to H_2_S, but in the absence of sulfur some species produce H_2_ as a major end product (Bálint et al., 2005; Kanai et al., 2005). Based on genome sequence analysis, all *Thermococcus* species have at least one NAD(P)H-dependent cytoplasmic hydrogenase and a ferredoxin (Fd)-dependent membrane hydrogenase that translocates H^+^ or Na^+^ across the membrane. *Thermococcus onnurineus* also produces H_2_ using a formate-dependent membrane hydrogenase (Kim et al., 2010; Bae et al., 2012) and a CO-dependent membrane hydrogenase (Kim et al., 2013). *Thermococcus paralvinellae* produced H_2_ when grown on both maltose and tryptone (0.5% wt vol^−1^ of each) without sulfur (Hensley et al., 2014). Its complete genome sequence contains seven hydrogenase gene clusters (Jung et al., 2014). Four of these clusters are putatively membrane bound and use ferredoxin, formate and CO as electron donors coupled with H^+^/Na^+^ translocation, and three are cytoplasmic and use NAD(P)H or coenzyme F_420_ as electron pairs (Figure 1). However, nothing is known about H_2_ production kinetics and enzyme activities in *T. paralvinellae* when grown on varying substrates or environmental conditions without sulfur.

Most of what is known about the central metabolism of *Thermococcus* has been obtained from studying *Pyrococcus furiosus* (see Adams, 1999; Verhees et al., 2003 for reviews), which like *Thermococcus* is a member of the *Thermococcaceae*. Glycolysis in both genera occurs via a modified Embden-Meyerhof pathway that uses ADP-dependent kinases (Kengen et al., 1994) and glyceraldehyde-3-phosphate:Fd oxidoreductase *in lieu* of glyceraldehyde-3-phosphate dehydrogenase and phosphoglycerate kinase (Mukund and Adams, 1995). Peptide catabolism occurs via peptide transamination and glutamate dehydrogenase to form 2-keto acids and reduction potential in the form of NAD(P)H (Figure 1; Robb et al., 1992). Pyruvate and other 2-keto acids are oxidized to acetate and other carboxylic acids plus CO_2_, reduced Fd, and ATP (Blamey and Adams, 1993; Schäfer et al., 1993; Heider et al., 1996; Mai and Adams, 1996). Alternatively, pyruvate can be reduced to alanine (Kengen and Stams, 1994), and the acyl-CoA products can be potentially reduced to alcohols (Basen et al., 2014) if alternative terminal electron acceptors are not available. *T. paralvinellae* produced ethanol and butanol when sulfur was in limited supply suggesting that it can also ferment when necessary (Ma et al., 1995).

Hydrogen-producing *Thermococcus* support the growth of hydrogenotrophic methanogens in the absence of other H_2_ sources through syntrophy (Bonch-Osmolovskaya and Stetter, 1991; Canganella and Jones, 1994; Davidova et al., 2012; Ver Eecke et al., 2012) and could be an important alternative source of energy in low-H_2_ environments. *Thermococcus* could also be used to consolidate wastewater organic compound remediation and heat treatment for pathogen removal in a single step to decrease costs while producing H_2_ as an energy product (Angenent et al., 2004; Bougrier et al., 2007; Girguis and Holden, 2012). In the U.S., nearly all food and agricultural waste enters landfills, which is the largest contributor of material entering these sites (Staley and Barlaz, 2009; Levis et al., 2010). It is increasingly being diverted to wastewater treatment facilities for anaerobic digestion to limit landfill growth and eutrophication (Diggelman and Ham, 2003; Lai et al., 2009). The waste contains high concentrations of organic compounds and often harmful chemicals such as antibiotics and hormones (Bougrier et al., 2007; Wang and Wan, 2009; Ntaikou et al., 2010) that foul wastewater treatment equipment leading to increasing restrictions on entering waste content (Lee et al., 2007). Biological pre-treatment of large organic molecules by fermentative organisms lowers the high organic carbon load in waste, lowers wastewater treatment costs, and can produce bioenergy to partially offset costs (Angenent et al., 2004; Wang and Wan, 2009; Ntaikou et al., 2010).

While the catabolic and energy generation pathways of *T. paralvinellae* are reasonably well established, its physiology, growth, and H_2_ production kinetics under normal and potentially adverse growth conditions are poorly understood. In this study, the growth and H_2_ production rates, the production of other metabolites, and the activities of 13 enzymes were measured from *T. paralvinellae* grown at 82°C on maltose, tryptone, and maltose plus tryptone as well as maltose plus acetate and maltose at low pH (pH 5) as potentially inhibitory conditions. *P. furiosus* was grown and analyzed in parallel at 95°C for comparison. These two organisms were grown for the first time on waste milk from cows treated for mastitis with Ceftiofur or from untreated cows, and separately on spent brewery grain to examine the feasibility of using them to rapidly remediate agricultural wastes. Ceftiofur is a heat-labile β-lactam that inhibits peptidoglycan synthesis in bacteria (Sunkara et al., 1999). We sought to remove organic compounds during growth and the pathogens and Ceftiofur from the milk by heat treatment during growth of *T. paralvinellae* with concomitant production of H_2_.

## Materials and methods

### Organisms used

*T. paralvinellae* ES1 (DSM 27261) was from our hyperthermophile culture collection. *P. furiosus* (DSM 3638) was obtained from the Deutsche Sammlung für Mikroorganismen und Zelkulturen (Braunschweig, Germany).

### Growth conditions

The base medium for growth was from Adams et al. (2001). Different carbon sources were added to the base medium solution, which were 0.5% (wt vol^−1^) maltose plus 0.01% (wt vol^−1^) yeast extract (enzymatic, Difco); 0.5% (wt vol^−1^) tryptone (Difco) plus 0.01% yeast extract; and 0.5% maltose, 0.05% tryptone plus 0.01% yeast extract. To examine the effect of acetate on cells, 10 mM acetate was added to 0.5% maltose plus 0.01% yeast extract medium. To examine growth at low pH, 0.5% maltose plus 0.01% yeast extract medium was pH balanced to 5.00 ± 0.05 at room temperature. All other media were pH balanced to 6.80 ± 0.05 at room temperature. For bottle experiments, these media (20 ml) were contained in 60 ml serum bottles with 1 atm of N_2_ in the headspace. Growth was also tested on 0.5% (wt vol^−1^) lactose plus 0.01% yeast extract in base medium as a follow-up to the waste milk growth experiments described below.

For the waste media experiments, one of three waste types was added as a carbon source to the base medium above. The milk medium contained 0.1, 1, or 10% (vol vol^−1^) waste milk obtained fresh from a local dairy farm (Barstow Farms, Hadley, MA). For *T. paralvinellae*, 20, 30, 50, and 70% waste milk were also examined. The milk was from cows with mastitis that had been treated with Ceftiofur within the previous 48 h and from healthy untreated cows. The grain medium contained 0.1, 1, or 10% (wt vol^−1^) spent brewery grain from a local brewery (Amherst Brewing Company, Amherst, MA). The initial chemical oxygen demand (COD) of 10% waste was 357 ± 6 g l^−1^ (mean ± standard error) in milk from untreated cows, 363 ± 3 g l^−1^ in milk from treated cows, and 334 ± 3 g l^−1^ in spent grain waste as determined from a COD HR test kit (Hach Company). Milk and grain that was not used within 12 h was frozen at −20°C. The waste media (50 ml) were contained in 120 ml serum bottles with 1 atm of N_2_ in the headspace.

All bottle cultures were incubated in a forced-air oven without stirring at 82°C for *T. paralvinellae* and 95°C for *P. furiosus.* The serum bottles were inoculated with a logarithmic growth-phase culture that had been grown and transferred at least three successive times in bottles on the medium used so that cultures were adapted to that medium. For the maltose-only, tryptone-only, maltose-tryptone, maltose-acetate, and low pH maltose media experiments, 12 serum bottles were inoculated concurrently and a pair of bottles was removed at different times during growth until the cultures reached stationary growth phase. For the waste media experiments, triplicate bottles were inoculated and subsampled every 12 h for up to 100 h. Cell concentrations were measured using a Petroff-Hausser counting chamber and phase-contrast light microscopy. The specific growth rate (μ) of the culture was determined by a best-fit curve through the logarithmic portion of the growth data.

Cultures were also grown in duplicate in a 20-L bioreactor for each defined media type and for 1% milk from Ceftiofur-treated cows. The media were flushed with N_2_:CO_2_ (80:20%) at a flow rate of 60 ml min^−1^, stirred at 120–150 rpm, and heated to 82 ± 0.1°C for *T. paralvinellae* and 95 ± 0.1°C for *P. furiosus*. CO_2_ was added to the flushing gas to help offset the CO_2_ lost from the NaHCO_3_ in the base medium. With the exception of the low pH condition (pH 5.0 ± 0.1), the pH of the bioreactor media was set at 6.8 at room temperature but rose with temperature to pH 7.2 where it was maintained (±0.1 pH unit) by the automatic addition of 5% (wt vol^−1^) NaHCO_3_ at the incubation temperature. Cells from the bioreactor were harvested when they reached late logarithmic growth phase. The medium was drained from the bioreactor through a glass cooling coil bathed in an ice-water-slurry into a carboy. The cells were then concentrated to less than 2 L by ultrafiltration using a hollow fiber cartridge (0.2-μm pore size; Amersham Biosciences) and further concentrated by centrifugation at 10,000 × *g* for 45 min. The exception was cells grown on the milk due to flocculation in the medium where only centrifugation was used to concentrate the cells. The resulting pellets were resuspended in an anoxic chamber in < 5 ml of degassed 50 mM Tris buffer (pH 8.0) plus 2 mM of sodium dithionite (DT), sealed in a N_2_-flushed serum bottle, and frozen at −20°C.

### Metabolite measurements

Metabolite measurements were only performed on bottle experiments. A subsample (100 μl) of headspace from each bottle at each time point was used to measure the amount of H_2_ present using a gas chromatograph (Shimadzu GC-8A) equipped with a thermal conductivity detector, a 60/80 Carboxen 1000 column (Supelco), and Ar as the carrier gas. Hydrogen yields per cell (*Y*_p∕x_) were determined by plotting the amount of H_2_ produced against cell concentrations for each set of time points within a growth curve. Specific H_2_ production rates (*q*) were calculated from (*Y*_p∕x_ × μ)/0.693, and theoretical maximum H_2_ production rates (*r*_max_) were calculated from the product of *q* times the maximum cell concentration (cell_max_). For defined media experiments, soluble metabolites were measured after 18 h of incubation. A 1.5 ml aliquot was transferred into an Eppendorf microfuge tube and spun at 14,000 rpm for 5 min. The supernatant was decanted and filtered through a 0.22 μm pore size filter (13 mm diameter, GVPP, Millipore) and 100 μl of 1 M H_2_SO_4_ was added to 1 ml of filtrate. Samples were run through an Aminex HPX-87H ion exclusion column (300 mm × 7.8 mm, Bio-Rad) with guard column (BioRad microguard Cation H) with 5 mM H_2_SO_4_ as the mobile phase using an ultra-pressure liquid chromatography (UPLC) system (Shimadzu) equipped with a refractive index detector. The column was kept at 30°C with a 0.6 ml min^−1^ flow rate and a 30 min sampling time.

### Waste remediation

To determine which portion of the milk waste was degraded during growth, 1% waste milk from Ceftiofur treated cows and untreated cows were inoculated separately with each organism and incubated as before along with uninoculated triplicate controls. A 1.5 ml liquid subsample was removed from each serum bottle every 12 h for soluble protein and reducing sugar measurements. For protein measurements, 1 ml of sample was filtered through a 0.22 μm pore size filter (13 mm diameter, GVPP, Millipore) and the protein in the filtrate was measured using a protein assay kit (Bio-Rad). For the reducing sugar measurements, 0.5 ml of sample and 0.5 ml of dinitrosalicylic acid solution (DNS) were heated to 100°C for 5 min, cooled in ice for 5 min, and then absorbance measured at 545 nm with lactose used as standard. The DNS was composed of the following (per liter): 7.06 g of 3,5-dinitrosalicylic acid, 13.2 g of sodium hydroxide, 204 g of Rochelle salt, 5.53 g of sodium metabisulfite, and 5.06 ml phenol (boiled). Uninoculated controls determined if indigenous microbes were affecting waste content or H_2_ production.

To determine if pathogens were removed from the milk, 1% waste milk from Ceftiofur-treated cows and untreated cows that was less than 48 h old, as well as base medium without an added carbon source, were plated via a dilution-to-extinction method onto Luria Bertani, Sheep's Blood, and MacConkey agar plates both prior to and after 100 h of incubation with *T. paralvinellae* at 82°C and were incubated up to 2 days at 37°C. Sheep's Blood agar is selective for Gram-positive bacteria, the most common causative agent of bovine mastitis, and MacConkey agar is selective for Gram-negative enteric bacteria, which are another source of mastitis. Technical triplicates of each media, time point, and agar plate type were run. The number of colony forming units per ml of inoculum was determined for all plates. To determine if Ceftiofur was present in waste milk, samples of undiluted waste milk from treated cows and samples of 10% milk media before and after 100 h of incubation at 82°C with and without inoculation with *T. paralvinellae* were tested using a Ceftiofur ELISA Test Kit (Bioo Scientific) as described by the manufacturer.

### Protein fractionation

All sample transfers and manipulations were carried out in an anoxic chamber and buffers were degassed with N_2_. Stored frozen cells were allowed to warm to room temperature and 2 μg ml^−1^ of DNase I were added. The cell suspension was mixed for 30 min. The sample vial was then placed in an ice-water slurry and sonicated for 30 min (Fisher Scientific, Sonic Dismembrator 500). Phase-contrast microscopy confirmed cell lysis. The cell suspension was spun in a centrifuge at 100,000 × *g* for 45 min. The supernatant was removed as the soluble protein fraction and the pellet was resuspended in buffer after being homogenized with a glass tissue grinder. The suspension was spun at 100,000 × *g* for 45 min as before and resuspended three times to wash the pellet. After the final spin, the pellet was resuspended in 1 ml of buffer. This was used as the insoluble protein fraction. The protein concentrations of the soluble protein fractions were determined spectrophotometrically using a protein assay kit (Bio-Rad). The protein concentrations of the insoluble protein fractions were determined using the DC Protein Assay kit (Bio-Rad). Bovine serum albumin was used as the standard for both procedures. Protein fractions that were not used immediately for enzyme activity assays were flash frozen in liquid N_2_ and stored at −80°C.

### Enzyme assays

Enzyme activities are expressed as units where 1 U is equal to 1 μmol of substrate transformed min^−1^. The buffer used for all assays was 100 mM EPPS buffer (pH 8.4) and the assay temperature was 80°C, unless otherwise noted. At least three technical replicates were run for each assay. For enzyme assays containing benzyl viologen (BV) or methyl viologen (MV), a trace amount of 2 mM sodium dithionite (DT) was added to slightly reduce the buffer. The amount of all reagents used is given as the final concentration in the reaction vial.

The following anaerobic enzyme activity assays were performed using the insoluble protein fraction and were contained in rubber stopper-sealed serum vials (8 ml volume) that had been degassed and flushed with N_2_. Membrane-bound hydrogenase (Mbh) (Sapra et al., 2000) activity was determined by following the H_2_ evolution rate in a discontinuous assay using 3 mM MV reduced with 30 mM DT as the electron donor. H_2_ was measured by gas chromatography as described above. Membrane-bound ATP synthase (ATPase) (Pisa et al., 2007) activity was determined by following the phosphate evolution rate in a discontinuous assay by adding 2.5 mM sodium ATP after 4 min of initial incubation to 100 mM MES-100 mM Tris buffer (pH 6.0) containing 5 mM MgCl_2_ and 200 mM KCl. The reaction was stopped after 2, 4, and 6 min by placing the serum vial on ice. The concentration of the inorganic phosphate produced was measured spectrophotometrically as described previously (Heinonen and Lahti, 1981).

The following anaerobic enzyme activity assays were measured spectrophotometrically using the soluble protein fraction and were contained in rubber stopper-sealed glass cuvettes that had been degassed and flushed with N_2_. Cytoplasmic hydrogenase (Sulf) (Bryant and Adams, 1989; Ma et al., 2000) activity was determined by measuring the reduction of 1 mM BV at 600 nm [ε = 7400 (M^•^cm)^−1^] under an H_2_:CO_2_ (80:20%) headspace. Pyruvate:Fd oxidoreductase (POR) (Blamey and Adams, 1993) and 2-ketoisovalerate:Fd oxidoreductase (VOR) (Heider et al., 1996) activities were determined by measuring the reduction of 1 mM MV at 600 nm [ε = 12,000 (M^•^cm)^−1^] and 1 mM BV, respectively, in an assay mixture that contained 2 mM MgCl_2_, 0.4 mM thiamine pyrophosphate (TPP), and 0.2 mM coenzyme A (CoA). Pyruvate (10 mM) and 5 mM 2-ketoisovalerate were used as the substrates, respectively. Aldehyde:Fd oxidoreductase (AOR) (Mukund and Adams, 1991) and formaldehyde:Fd oxidoreductase (FOR) (Roy et al., 1999) activities were determined by measuring the reduction of 3 mM BV using 0.5 mM crotonaldehyde and 0.25 mM formaldehyde, respectively, as the substrate. Fd:NAD(P)H oxidoreductase (FNOR) (Ma and Adams, 1994) activity was determined by measuring the reduction of 3 mM BV in 50 mM CAPS buffer (pH 10.3) using 0.4 mM NADPH as the substrate. Alcohol dehydrogenase (ADH) (Ma and Adams, 1999) was determined by measuring the reduction of 0.4 mM NADP^+^ at 340 nm [ε = 6220 (M^•^cm)^−1^] using 150 mM ethanol as the substrate. Formate dehydrogenase (FDH) (Ma et al., 1995) activity was determined by measuring the reduction of 5 mM BV at 600 nm in 100 mM EPPS (pH 8.4) using 10 mM sodium formate as the substrate. Alanine aminotransferase (AlaAT) (Ward et al., 2000) activity was determined by measuring the pyruvate evolution rate in a discontinuous assay by adding sample after 4 min of initial incubation to 100 mM KCl, 20 mM 2-ketoglutarate, 50 μM pyrodixal-5′-phosphate, and 50 mM L-alanine. The reaction was stopped after 2, 4, and 6 min by placing the serum vial on ice. The amount of pyruvate in each vial was determined aerobically at room temperature using a lactate dehydrogenase assay that contained 100 mM potassium phosphate (pH 7.0), 0.4 mM NADPH, and 50 U of LDH from rabbit muscle (Sigma-Aldrich).

The following aerobic enzyme activity assays were performed using the soluble protein fraction and were measured spectrophotometrically in glass cuvettes. Glutamate dehydrogenase (GDH) (Robb et al., 1992) activity was determined by measuring the reduction of 0.4 mM NADP^+^ at 340 nm [ε = 6220 (M^•^cm)^−1^] using 6 mM sodium glutamate as the substrate. ADP-forming acetyl-CoA synthetase (ACS) (Bräsen and Schönheit, 2004) activity was determined by measuring the production of DTNB-CoA by adding sample to 100 mM MOPS buffer (pH 7.0), 0.25 mM DTNB, 5 mM MgCl_2_, 1 mM ADP, 5 mM K_2_HPO_4_, and 0.2 mM acetyl-CoA.

### Statistical analyses

Results were subjected to statistical analyses in R (R Core Team, 2013). The growth rates, metabolite production rates, enzyme activities, and colony-forming-unit yields were compared using analysis of variance (ANOVA) and Tukey's Honestly Significant Difference test (α = 0.05). The mean of at least three replicates are reported as means ± standard error (SE).

## Results

### Growth and metabolite production

**Figure 2:**
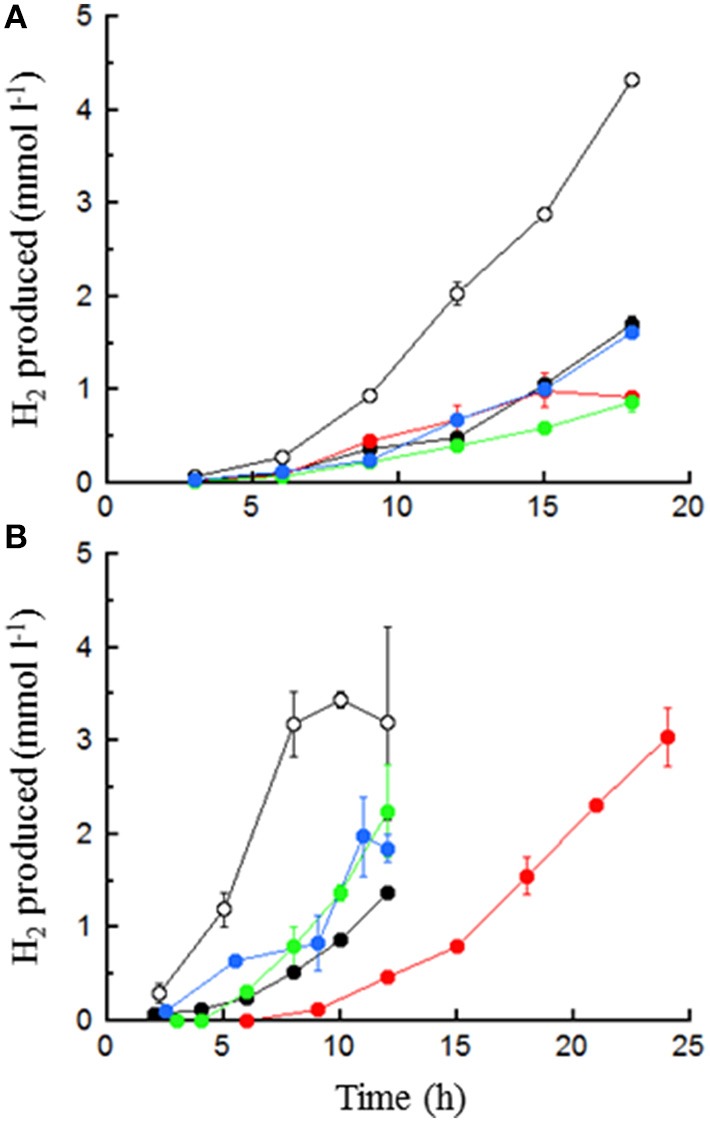
**H_**2**_ production by ***T. paralvinellae*** (A) and ***P. furiosus*** (B) in 0.5% maltose (○), 0.5% tryptone (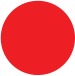), 0.5% maltose + 0.05% tryptone (●), 0.5% maltose + 10 mM acetate (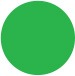), and 0.5% maltose at pH 5.0 (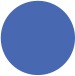)**. Error bars represent the standard error.

**Figure 3:**
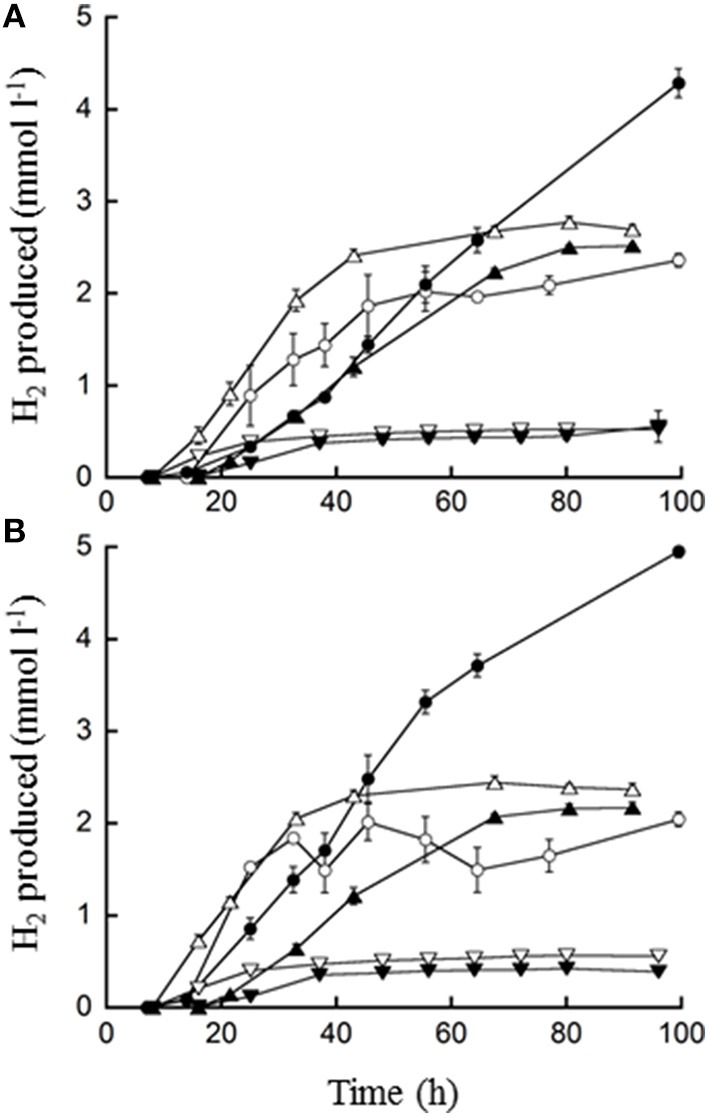
**H_2_ production by *T. paralvinellae* (filled symbols) and *P. furiosus* (open symbols) when grown on waste milk from cows treated with Ceftiofur (A) and from untreated cows (B)**. The concentrations of waste milk used were 10 (○, ●), 1 (Δ, ▲), and 0.1% (▽, ▼). Error bars represent the standard error.

**Figure 4:**
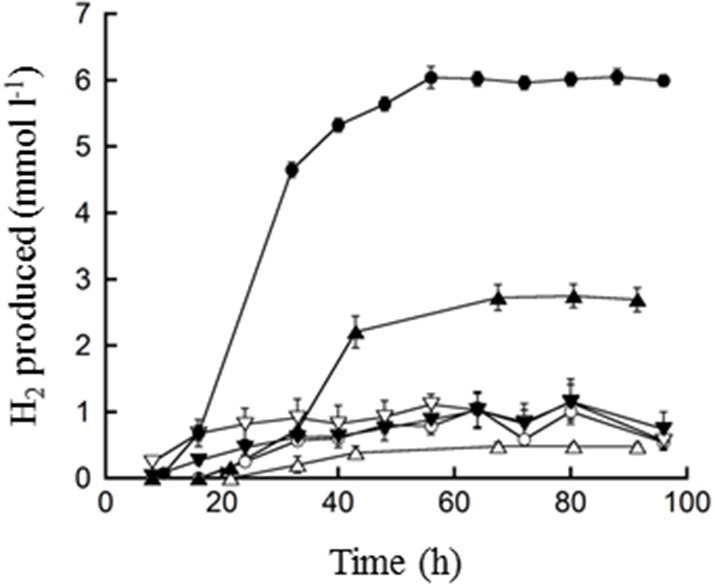
**H_2_ production by *T. paralvinellae* (filled symbols) and *P. furiosus* (open symbols) when grown on spent brewery grain**. The concentrations spent grain used were 10 (○, ●), 1 (Δ, ▲), and 0.1% (▽, ▼). Error bars represent the standard error.

Both *T. paralvinellae* and *P. furiosus* grew and produced H_2_ in the various maltose- and tryptone-containing media over 12–24 h (Figure 2), the milk waste media over 100 h (Figure 3) and the spent brewery grain media over 100 h (Figure 4). Neither organism grew on the 0.5% lactose plus 0.01% yeast extract medium. For both organisms, the maltose-tryptone medium was among those that produced the largest cell_max_ and *r*_max_ (Table 1). For *T. paralvinellae*, this condition was among those that also showed the largest *q* (specific H_2_ production rate) (F_4_ = 330.6, *p* < 0.001). While H_2_ was the primary metabolite produced, both organisms also produced acetate, butyrate, succinate, isovalerate, and formate (Table 2). Relative to H_2_ production, acetate production was highest in tryptone-only and maltose-acetate media for *T. paralvinellae* and in maltose-only medium for *P. furiosus*. Assuming four acetate molecules produced per molecule of maltose consumed, less than 0.5 mM maltose was used by either organism. Succinate, isovalerate and butyrate were also produced in various media (Table 2). *T. paralvinellae* produced formate when grown on maltose-only medium, while *P. furiosus* produced it in maltose-only and maltose-acetate media (Table 2). Ethanol was not detected in any medium tested for either organism.

**Table 1:** ***T. paralvinellae* and *P. furiosus* kinetic parameters**.

**Kinetic parameter^†^**	**Media type**					***p*-value**
	**0.5% maltose**	**0.5% tryptone**	**0.5% maltose + 0.05% tryptone**	**0.5% maltose + 10 mM acetate**	**0.5% maltose (pH 5)**	
***T. paralvinellae***						
μ (h^−1^)	0.45 ± 0.11	0.35 ± 0.21	0.34 ± 0.13	0.32 ± 0.08	0.35 ± 0.11	NS
cell_max_ (× 10^7^, ml^−1^)	13*^a^*	6*^b^*	13*^a^*	8*^b^*	5*^b^*	^*^
*Y*_p∕x_ (fmol H_2_ cell^−1^)	13 ± 3*^a^*	16 ± 5*^a^*	32 ± 7*^b^*	10 ± 1*^a^*	32 ± 8*^b^*	^***^
*q* (fmol H_2_ cell^−1^ h^−1^)	8 ± 3*^a^*	8 ± 3*^a^*	16 ± 5*^b^*	5 ± 1*^a^*	16 ± 4*^b^*	^***^
*r*_max_ (mmol H_2_ l^−1^ h^−1^)	1.1 ± 0.5*^a^^,^^b^*	0.5 ± 0.3*^a^*	2.0 ± 0.8*^b^*	0.4 ± 0.1*^a^*	0.8 ± 0.4*^a^*	^**^
***P. furiosus***						
μ (h^−1^)	0.43 ± 0.08	0.31 ± 0.22	0.49 ± 0.25	0.54 ± 0.13	0.39 ± 0.10	NS
cell_max_ (× 10^7^, ml^−1^)	10*^a^^,^^b^*	6*^b^*	19*^a^*	4*^b^*	8*^b^*	^**^
*Y*_p∕x_ (fmol H_2_ cell^−1^)	11 ± 2*^a^*	42 ± 11*^a^^,^^b^*	17 ± 3*^a^^,^^b^*	46 ± 29*^b^*	17 ± 8*^a^^,^^b^*	^**^
*q* (fmol H_2_ cell^−1^ h^−1^)	7 ± 1*^a^*	19 ± 6*^a^^,^^b^*	12 ± 4*^a^^,^^b^*	36 ± 24*^b^*	10 ± 5*^a^^,^^b^*	^**^
*r*_max_ (mmol H_2_ l^−1^ h^−1^)	0.7 ± 0.2*^a^*	1.1 ± 0.3*^a^*	2.3 ± 0.8*^b^*	1.4 ± 0.8*^a^^,^^b^*	0.7 ± 0.3*^a^*	^*^

† *The kinetic parameters are based on the best-fit line through the data (±95% confidence interval). μ, specific growth rate; cell_max_, maximum cell concentration; Y_p∕x_, product yield coefficient per cell; q, specific H_2_ production rate; r_max_, maximum H_2_ production rate. p-values calculated from one-way ANOVA test comparing results across treatments*.

* *p < 0.05*,

** p < 0.01, and

*** *p < 0.001*.

a, b *Tukey post-hoc test indicates groups whose members are not significantly different. NS, no significance*.

**Table 2:** **Mean product formation (mM, ± standard error) by *T. paralvinellae* and *P. furiosus* after 18 h of incubation in the defined media relative to uninoculated controls**.

**Product**	**0.5% maltose**	**0.5% tryptone**	**0.5% maltose + 0.05% tryptone**	**0.5% maltose + 10 mM acetate**	**0.5% maltose (pH 5)**
***T. paralvinellae***					
H_2_	0.47 ± 0.04	0.76 ± 0.02	1.18 ± 0.00	0.29 ± 0.01	0.05 ± 0.01
Acetate	0.20 ± 0.01	1.14 ± 0.09	0.30 ± 0.03	0.69 ± 0.00	0.01 ± 0.00
Butyrate	0	0	0	0.06 ± 0.00	0
Succinate	0	0.24 ± 0.01	0	0	0
Isovalerate	0	0.31 ± 0.00	0.02 ± 0.01	0	0
Formate	0	0.54 ± 0.06	0	0	0
***P. furiosus***					
H_2_	2.00 ± 0.00	1.71 ± 0.08	4.92 ± 0.47	5.51 ± 1.18	3.71 ± 0.16
Acetate	0.95 ± 0.05	0.06 ± 0.05	1.14 ± 0.27	1.66 ± 0.28	0.79 ± 0.06
Butyrate	0	0	0.01 ± 0.00	0	0
Succinate	0.14 ± 0.01	0.10 ± 0.00	0	0.22 ± 0.16	0
Isovalerate	0	0.46 ± 0.00	0.28 ± 0.28	0	0
Formate	2.14 ± 0.08	0	0.01 ± 0.01	1.51 ± 0.25	0

### Growth and H_2_ production in waste media

**Figure 5:**
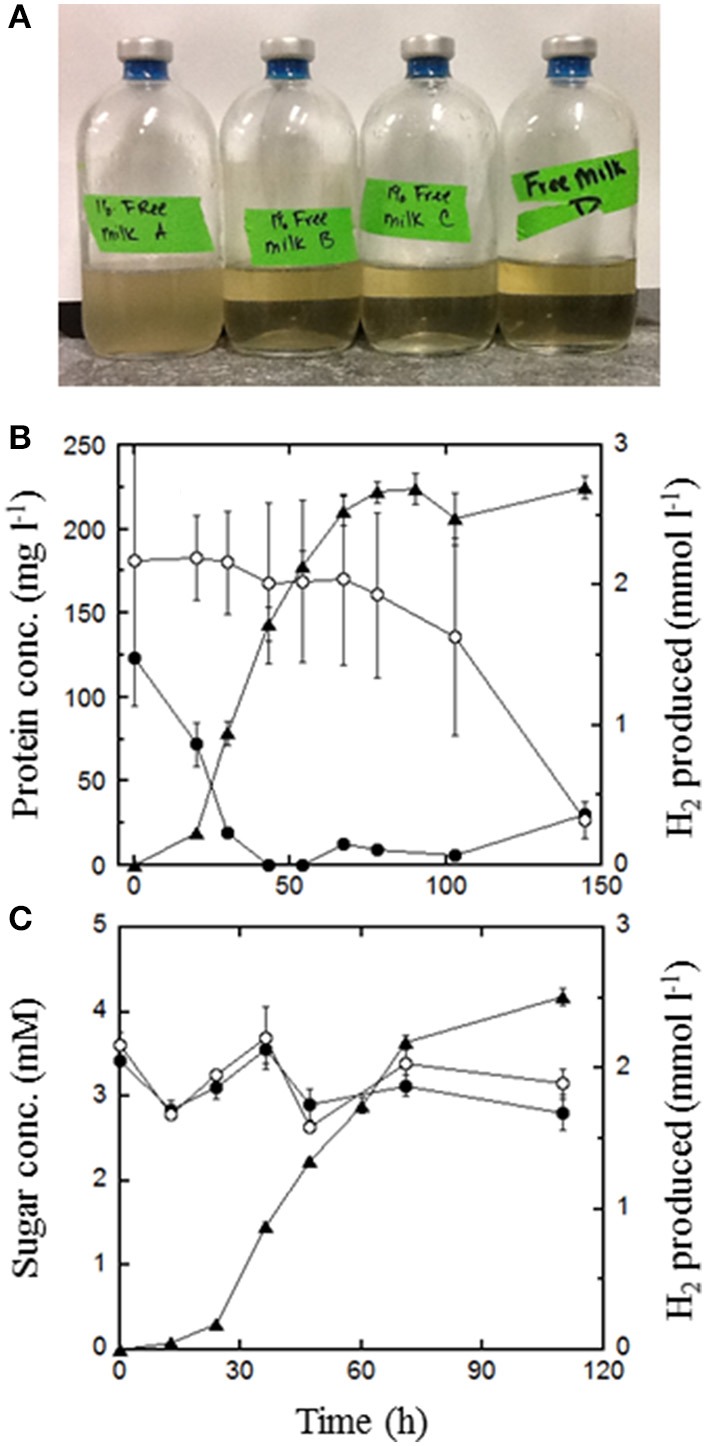
**Degradation of waste milk by *T. paralvinellae* during 100 h of incubation at 82°C. (A)** Clarification of media containing 1% waste milk from untreated cows following 100 h incubation. The bottle on the left was an uninoculated control. Concentrations of protein **(B)** and reducing sugars **(C)** in 1% waste milk from untreated cows. The data shown are from inoculated bottles (●) and from uninoculated controls (○) as well as H_2_ produced from inoculated bottles (▲). There was no H_2_ production in uninoculated bottles. Error bars represent the standard error.

*T. paralvinellae* produced increasingly higher amounts of H_2_ with increasing concentration of waste milk for both milk types (Figure 3). The increase in H_2_ production continued with increasing milk concentration up to the highest concentration tested (70%; data not shown). For *P. furiosus*, the amount of H_2_ produced from 1% waste milk was higher than the amount for both 0.1 and 10% waste milk (Figure 3). *T. paralvinellae* produced more H_2_ than *P. furiosus* in all waste conditions tested except 0.1% milk from Ceftiofur-treated cows and 1% milk from untreated cows. Growth and H_2_ production did not significantly change for either microorganism between waste milk from cows treated with Ceftiofur and from untreated cows. There was visual clarification by eye of waste milk media following 100 h of incubation at 82°C with *T. paralvinellae*, but not in the uninoculated control bottle incubated at the same temperature (Figure 5A). When 1% waste milk was incubated with *T. paralvinellae*, the concentration of soluble protein decreased to undetectable concentrations within 48 h and was significantly lower (*p* < 0.01) than the soluble protein concentrations in uninoculated bottles (Figure 5B). The amount of reducing sugars in the milk (Figure 5C) and the total COD decreased over time, but the concentrations were not significantly different from those in uninoculated bottles. Cell counts were highly variable when grown on milk due to precipitation and clumping of the milk at high temperatures.

*T. paralvinellae* produced increasing amounts of H_2_ (up to 6 mmol l^−1^) with increasing amounts of spent brewery grain as the feedstock (Figure 4). In contrast, *P. furiosus* produced less than 1 mmol of H_2_ l^−1^ on 0.1, 1, and 10% spent brewery grain (Figure 4). Microscopically, neither *T. paralvinellae* nor *P. furiosus* were preferentially associated with the cellulose fibers of the spent brewery grain, but could not be accurately counted due to the presence of the grain.

### Remediation of waste milk

Dilution-to-extinction plating of 1% milk showed that milk from Ceftiofur-treated cows and untreated cows contain similar concentrations of bacteria prior to incubation (Table 3). The exception was that there were colony-forming units found on MacConkey agar plates, which is selective for Gram-negative enteric bacteria, from untreated cows that were completely absent from plates from cows treated with Ceftiofur. Also, the concentrations of λ colonies on Sheep's Blood agar plates, which can represent *Staphylococcus* spp. (the typical cause of mastitis), were higher in cows being treated for mastitis. All plate types showed no colony forming units from medium that had been incubated with *T. paralvinellae* for 100 h at 82°C (Table 3). The concentration of Ceftiofur in waste milk from a cow treated within 48 h of milking was 25.7 ± 0.2 ng ml^−1^ (mean ± standard error). With and without inoculation with *T. paralvinellae*, the amount of Ceftiofur in 10% waste milk medium was below the detection limit within an hour of incubation at 82°C.

**Table 3:** **Mean colony forming units (±standard error, *n* = 3) per ml of fresh (< 2 h old) 1% (vol vol^−1^) waste milk from Ceftiofur-treated and untreated cows in base medium, and base medium without a carbon source**.

**Treatment**	**Luria Broth (× 10^3^)**	**Sheep's blood**			**MacConkey**	
		**α (× 10^2^)**	**β (× 10^2^)**	**λ (× 10^2^)**	**Fermenter**	**Non-fermenter**
**WITHOUT HEAT TREATMENT**						
Ceftiofur-treated	5.7 ± 4.2	7.0 ± 3.0	2.0 ± 0.3	93 ± 77	0	0
Untreated	4.4 ± 3.7	4.3 ± 3.8	4.6 ± 2.8	2.6 ± 1.2	3 ± 3	66 ± 56
Control	0	0	0	0	0	0
**100 h at 82**°**C**						
Ceftiofur-treated	0	0	0	0	0	0
Untreated	0	0	0	0	0	0
Control	0	0	0	0	0	0

### Growth in a 20-l bioreactor

**Figure 6:**
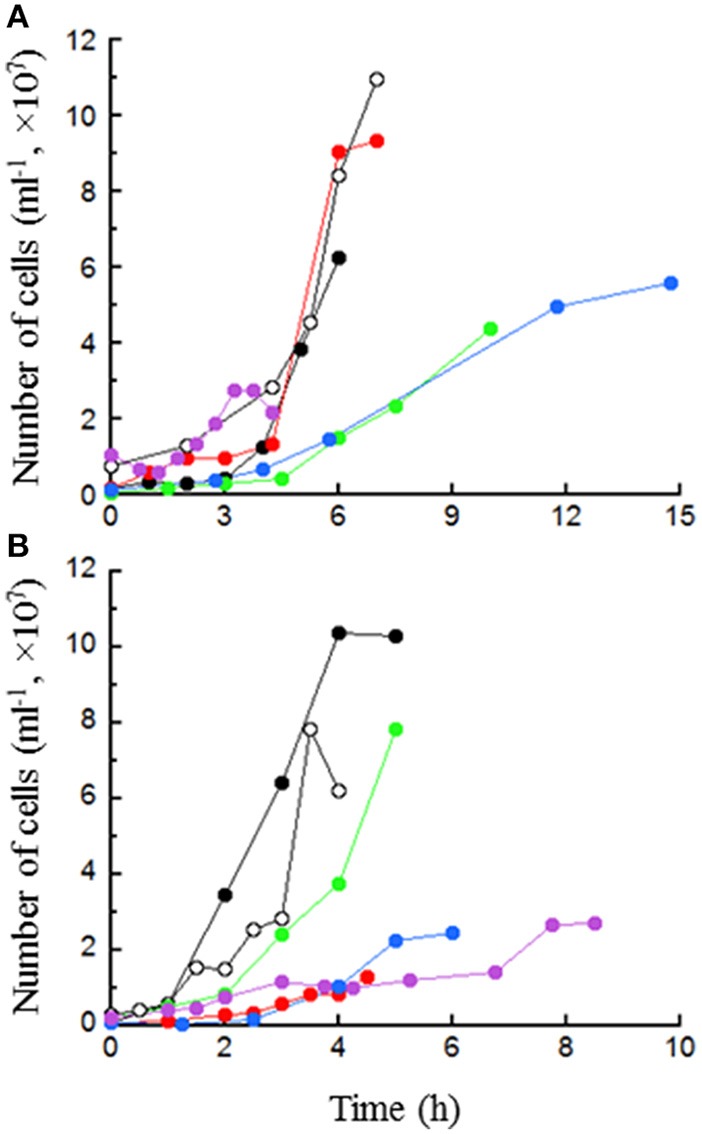
**Growth of *T. paralvinellae* (A) and *P. furiosus* (B) in the 20-l bioreactor in 0.5% maltose (●), 0.5% tryptone (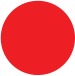), 0.5% maltose + 0.05% tryptone (○), 0.5% maltose + 10 mM acetate (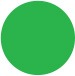), 0.5% maltose at pH 5.0 (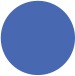), and 0.1% waste milk (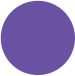)**. Data are examples from individual bioreactor reactions.

The growth rates of *T. paralvinellae* in the bioreactor were 0.67 ± 0.06 h^−1^ (mean ± standard error) on maltose only (pH 6.8), 0.59 ± 0.05 h^−1^ on tryptone only, 0.53 ± 0.14 h^−1^ on maltose plus tryptone, 0.38 ± 0.05 h^−1^ on maltose plus acetate, 0.36 ± 0.04 h^−1^ on maltose only (pH 5.0), and 0.49 ± 0.09 h^−1^ on milk containing Ceftiofur (Figure 6A). For *T. paralvinellae*, specific growth rates increased in the bioreactor relative to bottles when the cultures were grown on maltose and maltose-tryptone media. Otherwise, specific growth rates on the other three defined media were unchanged. *T. paralvinellae* growth rates on maltose at pH 5 or in the presence of acetate were lower in the bioreactor than maltose only in the bioreactor suggesting that pH and acetate can have some inhibitory effect in a bioreactor. Specific growth rates of *P. furiosus* on each of the media increased significantly in the 20-l bioreactor relative to growth in serum bottles (Figure 6B). The growth rates of *P. furiosus* in the bioreactor were 0.95 ± 0.08 h^−1^ (mean ± standard error) on maltose only (pH 6.8), 0.73 ± 0.04 h^−1^ on tryptone only, 0.73 ± 0.05 h^−1^ on maltose plus tryptone, 0.67 ± 0.06 h^−1^ on maltose plus acetate, 0.71 ± 0.02 h^−1^ on maltose only (pH 5.0), and 0.29 ± 0.03 h^−1^ on milk with Ceftiofur. As seen in the bottles, the maximum cell concentration of *P. furiosus* in the bioreactor on peptide medium was much lower than rates on maltose and maltose-tryptone media, while there was no difference in the growth rates in these media with *T. paralvinellae*.

### Enzyme activities

A summary of all specific enzyme activities are provided in Table 4. Overall, enzymatic activities in both organisms changed the most when cells were grown under stressful conditions. *T. paralvinellae* and *P. furiosus* showed the largest dissimilarity in enzyme activities when grown on maltose-acetate medium (11 of 13 enzymes for both organisms) and low pH maltose medium (6 of 13 activities for *T. paralvinellae* and 2 of 13 enzymes for *P. furiosus*). For *T. paralvinellae*, membrane-bound ATP synthase, GDH, and AlaAT activities were higher than all other enzyme activities. For *P. furiosus*, Mbh activities were higher than any other enzyme activity for all defined media except for FNOR activity which was higher when cells were grown on maltose-only medium and GDH activity which was higher when cells were grown on tryptone-only medium.

**Table 4:** ***T. paralvinellae* and *P. furiosus* specific enzyme activities**.

**Enzyme^†^**	**Media type**						***p*-value**
	**0.5% maltose**	**0.5% tryptone**	**0.5% maltose** **0.05% tryptone**	**0.5% maltose +** **10 mM acetate**	**0.5% maltose (pH 5)**	**1% waste milk**	
***T. paralvinellae***							
Mbh	3.34 ± 0.91	4.62 ± 2.20	1.53 ± 0.31	1.36 ± 0.29	0.18 ± 0.18	ND	NS
Sulf	0.21 ± 0.03*^a^*	0.54 ± 0.20*^a^*	1.53 ± 0.15	0.06 ± 0.01*^a^*	0.05 ± 0.01*^a^*	0.35 ± 0.14*^a^*	^***^
ATPase	0.23 ± 0.04*^a^^,^^b^*	3.30 ± 0.44*^d^*	0.39 ± 0.29*^a^^,^^c^*	4.41 ± 0.03*^d^*	1.33 ± 0.20*^b^^,^^c^*	ND	^***^
FNOR	0.21 ± 0.07*^a^*	3.42 ± 0.16*^c^^,^^d^*	3.33 ± 0.60*^b^^,^^c^*	9.10 ± 1.02	1.84 ± 0.22*^a^^,^^b^^,^^d^*	1.71 ± 0.22*^a^^,^^b^^,^^d^*	^***^
GDH	12.09 ± 0.75*^a^*	9.03 ± 0.56*^a^^,^^c^*	3.88 ± 1.26*^b^*	3.89 ± 0.91*^b^*	8.31 ± 0.30*^c^*	0.53 ± 0.14*^b^*	^***^
AlaAT	1.21 ± 0.47*^a^*	5.80 ± 2.46*^a^^,^^b^*	5.08 ± 1.27*^a^^,^^b^*	3.01 ± 0.11*^a^^,^^b^*	8.00 ± 0.37*^b^*	2.42 ± 0.45*^a^^,^^b^*	^*^
POR	2.59 ± 0.13*^a^*	0.46 ± 0.10*^b^*	1.29 ± 0.09	0.41 ± 0.05*^b^*	2.16 ± 0.40*^a^*	0.18 ± 0.05*^b^*	^***^
VOR	0.34 ± 0.07*^a^*	0.11 ± 0.03*^b^*	0.44 ± 0.05*^a^*	0.02 ± 0.01*^b^*	0.11 ± 0.03*^b^*	0.14 ± 0.03*^b^*	^***^
ACS	0.34 ± 0.07*^a^*	0.52 ± 0.02*^b^*	0.60 ± 0.03*^b^*	0.21 ± 0.02*^a^^,^^c^*	0.60 ± 0.02*^b^*	0.13 ± 0.06*^c^*	^***^
AOR	0.35 ± 0.05*^a^*	0.48 ± 0.09*^a^*	0.29 ± 0.05*^a^*	0.60 ± 0.10*^a^*	1.55 ± 0.43	0.10 ± 0.03*^a^*	^***^
FOR	0.28 ± 0.10*^a^^,^^b^*	0.62 ± 0.14*^b^*	0.12 ± 0.03*^a^*	0.34 ± 0.13*^a^^,^^b^*	0.46 ± 0.06*^a^^,^^b^*	0.16 ± 0.02*^a^*	^**^
ADH	0.03 ± 0.01*^a^^,^^b^*	0.02 ± 0.00*^a^^,^^b^*	0.07 ± 0.03*^a^*	0*^b^*	0.02 ± 0.01*^a^^,^^b^*	0*^b^*	^**^
FDH	0.06 ± 0.02*^a^*	0.03 ± 0.00*^a^^,^^b^*	0.02 ± 0.00*^a^^,^^b^*	0.04 ± 0.01*^a^^,^^b^*	0.02 ± 0.00*^a^^,^^b^*	0.01 ± 0.01*^b^*	^*^
***P. furiosus***							
Mbh	4.99 ± 0.31*^a^*	9.55 ± 0.50*^a^^,^^b^*	12.74 ± 0.24*^b^*	11.42 ± 0.19*^b^*	5.39 ± 0.40*^a^^,^^b^*	ND	^**^
Sulf	1.27 ± 0.11*^a^^,^^a^^,^^c^*	0.73 ± 0.19*^a^^,^^a^^,^^d^*	3.44 ± 0.49	1.48 ± 0.29*^a^^,^^a^^,^^e^*	0.15 ± 0.05*^d^^,^^f^*	0.52 ± 0.02*^c^^,^^c^^,^^f^*	^***^
ATPase	0.05 ± 0.00*^a^*	0.01 ± 0.01*^a^*	0.70 ± 0.22	0*^a^*	0*^a^*	ND	^*^
FNOR	6.10 ± 0.60*^a^*	3.90 ± 0.32*^a^^,^^b^*	4.59 ± 0.48*^a^*	3.83 ± 0.47*^a^^,^^b^*	0.31 ± 0.12*^b^*	3.16 ± 1.59*^a^^,^^b^*	^***^
GDH	1.85 ± 0.36*^a^*	9.90 ± 1.08*^c^*	4.69 ± 0.42*^b^*	0.69 ± 0.08*^a^*	0.91 ± 0.09*^a^*	0.68 ± 0.16*^a^*	^***^
AlaAT	0.54 ± 0.25	3.05 ± 0.61	2.59 ± 1.76	0.72 ± 0.14	0.36 ± 0.05	0.54 ± 0.29	NS
POR	2.27 ± 0.43	0.93 ± 0.10*^a^*	0.72 ± 0.03*^a^*	0.85 ± 0.09*^a^*	0.30 ± 0.02*^a^*	0.01 ± 0.01*^a^*	^***^
VOR	1.88 ± 0.23*^a^*	2.01 ± 0.45*^a^*	0.56 ± 0.12*^b^^,^^c^*	0.56 ± 0.06*^b^^,^^d^*	1.35 ± 0.05*^a^^,^^a^^,^^d^*	0.11 ± 0.05*^c^^,^^d^*	^***^
ACS	0.87 ± 0.09*^a^^,^^a^^,^^c^*	1.30 ± 0.12*^b^*	1.15 ± 0.24*^a^*	0.68 ± 0.09*^a^^,^^c^*	0.57 ± 0.04*^a^^,^^c^*	0.44 ± 0.19*^c^*	^***^
AOR	0.36 ± 0.04*^a^*	0.12 ± 0.03*^b^^,^^c^^,^^d^*	0.24 ± 0.01*^a^^,^^b^^,^^d^*	0.26 ± 0.07*^a^^,^^c^*	0.08 ± 0.02*^d^*	0.06 ± 0.02*^d^*	^***^
FOR	0.47 ± 0.08	0.27 ± 0.06	0.36 ± 0.01	0.45 ± 0.11	0.31 ± 0.09	0.47 ± 0.23	NS
ADH	0	0	0.01 ± 0.00	0	0	0.01 ± 0.01	NS
FDH	0.02 ± 0.00	0.06 ± 0.02	0.02 ± 0.01	0.01 ± 0.01	0.03 ± 0.01	0	NS

† *The specific activities (U [mg protein]^−1^) are the averages of no less than three technical replicates and two biological replicates (±standard error). ND, a specific activity was not determined. p-values calculated from one-way ANOVA test comparing enzymatic activity across treatments*.

* *p < 0.05*,

** p < 0.01, and

*** *p < 0.001*.

a, b, c, d, e, f *Tukey post-hoc test indicates groups whose members are not significantly different. NS, no significance*.

Mbh and ATP synthase activities could not be measured from cells grown on milk due to co-precipitation of insoluble cell fractions with heat curdled milk. The Mbh activities in *T. paralvinellae* did not show any significant change between the defined media. In contrast, Mbh activity in *P. furiosus* cells grown in maltose-tryptone medium was higher (F_4_ = 12.05, *p* < 0.05) than activities in cells from maltose and low pH maltose media, and activity in cells from maltose-acetate medium was higher than that in cells from maltose medium. Mbh activities in *P. furiosus* were higher than those in *T. paralvinellae* for cells grown on maltose-tryptone (F_1_ = 798, *p* < 0.001), maltose-acetate (F_1_ = 847, *p* < 0.005), and low pH maltose (F_1_ = 142.9, *p* < 0.01) media. Sulf activities from maltose-tryptone medium were higher in *T. paralvinellae* and *P. furiosus* cells than activities in cells from the other four defined media and 1% milk medium (F_5_ = 23.2, *p* < 0.001). Sulf activities were higher in *P. furiosus* than in *T. paralvinellae* for cells grown on maltose (F_1_ = 66.7, *p* < 0.001), maltose-tryptone (F_1_ = 14.5, *p* < 0.01), and maltose-acetate (F_1_ = 20.5, *p* < 0.01) media. Membrane-bound ATP synthase activity in *T. paralvinellae* was higher in cells grown on maltose-acetate and tryptone media than on the other three defined media (F_4_ = 54.2, *p* < 0.001). In *P. furiosus*, ATP synthase activity in cells grown on maltose-tryptone medium was higher than activities in cells from the other four defined media (F_4_ = 10.4, *p* < 0.02). Membrane-bound ATP synthase activity was higher in *T. paralvinellae* than in *P. furiosus* for cells grown on tryptone (F_1_ = 57.0, *p* < 0.02), maltose-acetate (F_1_ = 2.1 × 10^4^, *p* < 0.001), and low pH maltose (F_1_ = 44.4, *p* < 0.05) media.

In *T. paralvinellae*, AOR and FNOR activities were higher in cells grown on maltose-acetate medium than any of the other four defined media and 1% waste milk medium (F_5_ = 7.75, *p* < 0.001 and F_5_ = 37.72, *p* < 0.001, respectively). FNOR activity was lower in cells grown on maltose medium than on maltose-tryptone and tryptone media (F_5_ = 37.73, *p* < 0.01). VOR activity was higher in cells grown on maltose and maltose-tryptone media than any other media (F_5_ = 14.45, *p* < 0.05), POR activity was higher in cells grown on maltose and low pH maltose media than any other media (F_5_ = 30.32, *p* < 0.05), and GDH activity was higher (F_5_ = 28.8, *p* < 0.02) in cells grown on maltose medium than cells grown on low pH maltose, maltose-acetate, maltose-tryptone, and 1% milk media. In *P. furiosus*, GDH activity was higher in cells grown on tryptone medium than any of the other four defined media and 1% waste milk (F_5_ = 50.0, *p* < 0.001). POR activity was higher in cells grown on maltose medium than any of the other four defined media and 1% waste milk (F_5_ = 11.28, *p* < 0.001). The other enzyme activities for both organisms either did not change with growth medium or did not show a consistent pattern in their differences.

## Discussion

### Growth, H_2_ production kinetics, and enzyme activities

*T. paralvinellae* ES1 was isolated from a polychaete worm collected from a deep-sea hydrothermal vent in the northeastern Pacific Ocean and is commonly associated with growth on peptides and sulfur to produce H_2_S (Pledger and Baross, 1989). However, *T. paralvinellae* possesses seven hydrogenase gene clusters (Jung et al., 2014) and produces H_2_ *in lieu* of H_2_S when sulfur is omitted from the medium (Hensley et al., 2014). In this study, *T. paralvinellae* produced H_2_ when grown without sulfur on either a carbohydrate (maltose) or on peptides and under potentially inhibiting conditions such as high acetate concentrations and at pH 5. The maltose-tryptone medium improved H_2_ production for *T. paralvinellae* (Figure 2) rather than have no effect or a negative effect as observed with most mesophilic and thermophilic anaerobes (Kim et al., 2004; Pawar and van Niel, 2013). In many other H_2_ producing organisms, as the pH decreases H_2_ production becomes increasingly inhibited due to the effect of low pH on hydrogenases (Dabrock et al., 1992). While pH 5 did decrease the specific activity of many enzymes in *T. paralvinellae* and *P. furiosus*, it did not decrease the growth or H_2_ production rates relative to growth on maltose at pH 6.8. It was previously reported that the addition of 30 and 60 mM acetate did not affect the growth of *P. furiosus* (Krahe et al., 1996), but this is the first study to measure the impact of acetate on H_2_ production kinetics and enzyme activities. *T. paralvinellae* grew and produced H_2_ at rates and yields that were comparable to those of *P. furiosus* without sulfur on all media types tested. These data demonstrate that *T. paralvinellae* is amenable to producing H_2_ on diverse substrates and conditions.

Although the H_2_ production yields (*Y*_*p*∕*x*_) for the defined media were generally similar between *T. paralvinellae* and *P. furiosus*, the MV-dependent Mbh activity was lower in *T. paralvinellae* than in *P. furiosus* for most media. *T. paralvinellae* also produced larger amounts of H_2_ than *P. furiosus* when grown on high concentrations of wastes. These results may be due to differing H_2_ production pathways in the two organisms. *T. paralvinellae* possesses genes for putative CO-, F_420_-, and formate-dependent hydrogenases that are lacking in *P. furiosus*, in addition to the Mbh and Sulf in both organisms (Jung et al., 2014). If active, they may ameliorate possible H_2_ inhibition by having a more diverse electron carrier pool to draw from. For *T. paralvinellae*, POR, VOR, AOR, and GDH activities were significantly higher and FNOR lower when grown on maltose-containing media relative to the other media. Enzyme activities and metabolite production in *P. furiosus* were largely as previously described (Adams et al., 2001; Schut et al., 2003). POR activity in *P. furiosus* was significantly higher in cells grown on maltose and GDH activity was significantly higher in cells grown on peptides. This pattern differs somewhat from that seen in *T. paralvinellae* suggesting that redox balancing in *T. paralvinellae* might involve factors not found in *P. furiosus*.

As expected for both organisms, H_2_:acetate ratios were approximately two when cultures were grown on maltose-only medium, and isovalerate was only produced in tryptone-containing media. Final acetate concentrations were comparable to those reported previously for *Pyrococcus* and *Thermococcus* species grown in batch culture (Schäfer and Schönheit, 1992; Ma et al., 1995; Nohara et al., 2014). Alanine, ethanol, butanol, and formate are other metabolites that have been reported for various *Thermococcus* and *Pyrococcus* species when terminal electron acceptors are in limited supply or when the H_2_ partial pressure is elevated (Kengen and Stams, 1994; Ma et al., 1995; Nohara et al., 2014). Alcohol production and ADH and FDH activities increased in *T. paralvinellae* when it was grown on low (1 g l^−1^) sulfur concentrations (Ma et al., 1995). Recently, it was shown that *P. furiosus* reduces acetate and other carboxylic acids first to an aldehyde by AOR and then to an alcohol when a recombinant *adh*A gene is introduced into the organism's genome (Basen et al., 2014). The genomes of *T. paralvinellae* and *P. furiosus* contain four and two *adh*A genes, respectively (Robb et al., 2001; Jung et al., 2014), suggesting that they naturally have the ability to produce alcohols from aldehydes. However, alcohols were undetectable in this study and ADH activities were very low to undetectable in both organisms suggesting that alcohol production is not a significant alternative pathway for electron disposal for these organisms even in the presence of high acetate concentrations or low pH. Formate was produced by both organisms, sometimes surpassing acetate production, but FDH activities were low in both organisms and the mechanism for its production is unknown. Formate has not been reported previously for *P. furiosus*, although this may be for analytical reasons since previous detection methods were not focused on formate. Formate has been reported as a metabolite for *Thermococcus kodakarensis* (Nohara et al., 2014).

### Growth, H_2_ production kinetics, and enzyme activities

Over the past decade, food waste in the U.S. has risen to account for the largest proportion (21%) of municipal solid waste (MSW; Staley and Barlaz, 2009; United States Environmental Protection Agency (US EPA), 2014). As of 2012, only 3% of food waste in the U.S. was treated with the rest becoming a part of MSW and buried in landfills (United States Environmental Protection Agency (US EPA), 2014). Food waste is produced at every stage of production from farms and food processing facilities as pre-consumer waste to domestic waste (Lin et al., 2013). Although the amount of waste for each sector is fairly evenly spread, waste produced by the agricultural and manufacturing sector is generated in a more concentrated manner and would therefore be easier to collect, process, and treat to keep it from entering landfills and help offset the costs of collection. Therefore, recent developments in waste management technology have focused on pre-consumer waste. Diverting food waste to composting and biological treatment is considered promising to limit landfill growth and produce biofuels (Hermann et al., 2011). Microorganisms that catabolize long-chain carbohydrates and polypeptides and produce an energy product such as H_2_, CH_4_, and alcohols are ideal for these processes (Angenent et al., 2004; Alper and Stephanopoulos, 2009). This study showed that *T. paralvinellae* and *P. furiosus* grew and produced H_2_ on waste milk from cows treated with Ceftiofur and from healthy untreated cows and spent brewery grain. *T. paralvinellae* produced more H_2_ and grew on higher feedstock concentrations relative to *P. furiosus*, but generally grew slower than *P. furiosus* on the wastes.

Waste milk from dairy farms and cows being treated for mastitis (a commonly occurring infection and inflammation of utters) with the bacterial antibiotic Ceftiofur is particularly problematic. Cows undergoing treatment need to be milked daily and produce ~37 L of milk per cow per day. While only 0.1% of the initial antibiotic dose is excreted in milk, the milk cannot be added to the food supply for 5 days after treatment (Hornish and Kotarski, 2002). The U.S Food and Drug Administration requires that the concentration of Ceftiofur in waste milk be less than 50 ppb before disposal (Samanidou and Nisyriou, 2008). Because of the antibiotic, waste milk cannot be disposed of with MSW or sewage, nor should it be mixed with manure and spread onto fields as fertilizer due to increasing antibiotic resistance in nature. The waste milk is often pasteurized and fed to calves; however, resistance of calf gut bacteria to antibiotics was shown to increase in calves fed milk with increasing concentrations of antibiotics (Langford et al., 2003). Therefore, an alternative waste disposal mechanism is needed. Ceftiofur is a β-lactam that inhibits peptidoglycan synthesis and thus does not affect *Thermococcus* and *Pyrococcus* since they are archaea. In this study, there was no change in either growth rate or H_2_ production for either *T. paralvinellae* or *P. furiosus* when they were grown on milk from Ceftiofur-treated cows relative to milk from untreated cows indicating that the antibiotic was ineffective against them. Furthermore, the half-life of Ceftiofur at 67°C is 6 h, which decreases with increasing temperature (Sunkara et al., 1999). In our study, the concentration of Ceftiofur after 1 h of incubation at 82°C, with and without *T. paralvinellae*, was below the detection limit (~2 ppb).

Raw bovine milk contains by weight 3% protein (mostly casein), 4% fat, and 5% carbohydrate (mostly lactose; Wong et al., 1988). The solids in spent brewery grain are mostly carbohydrate (starch). In this study, *T. paralvinellae* and *P. furiosus* grew fastest and produced the most H_2_ on media containing a combination of carbohydrate and peptides. Soluble protein was removed from the milk by the organisms, but neither organism grew on lactose or removed it from the milk. They both possess genes for a β-galactosidase (Robb et al., 2001; Jung et al., 2014), for which the recombinant version of the enzyme from *P. furiosus* cleaved lactose (Dong et al., 2014). They also grow on cellobiose (Oslowski et al., 2011), which chemically is similar to lactose, suggesting that they might be adaptable to growth on lactose as well.

The only previous study to examine agricultural waste degradation linked with H_2_ production by a hyperthermophile was a two-stage process for keratin degradation. In the first stage, a *Bacillus* species was used to aerobically degrade feather waste into a keratin hydrolysate, which was then used in the second stage as a feedstock for H_2_ production by *Thermococccus litoralis* (Bálint et al., 2005). *T. litoralis* produced up to 3 mmol L^−1^ of H_2_ on that feedstock. Genetically modified *T. onnurineus* converted the CO in steel industry syngas into H_2_ three times faster than wild-type cells at rates up to 60 mmol L^−1^ h^−1^ demonstrating the potential for enhancing waste conversion rates through genetic engineering (Kim et al., 2013). In this study, *T. paralvinellae* degraded waste milk and spent brewery grain in a single consolidated processing step. The heat from the incubation eliminated the pathogens present and degraded the heat-labile antibiotic present in the waste milk. *T. paralvinellae* produced increasing amounts of H_2_ from both agricultural wastes types with increasing concentration. It also grows over a lower temperature range than *P. furiosus*, which may give *T. paralvinellae* a cost advantage over *P. furiosus* due to lower reactor heating costs. Therefore, it may be feasible to use *T. paralvinellae*, or a similar hyperthermophilic heterotroph, for rapid, mesoscale treatment of organic wastes to reduce the organic load, generate H_2_ as an energy byproduct, and remove pathogens.

## Author contributions

All authors listed, have made substantial, direct and intellectual contribution to the work, and approved it for publication.

### Conflict of interest statement

The authors declare that the research was conducted in the absence of any commercial or financial relationships that could be construed as a potential conflict of interest.
